# Improved up-and-down procedure for acute toxicity measurement with reliable LD_50_ verified by typical toxic alkaloids and modified Karber method

**DOI:** 10.1186/s40360-021-00541-7

**Published:** 2022-01-04

**Authors:** Yan-Yu Zhang, Yu-Feng Huang, Jie Liang, Hua Zhou

**Affiliations:** 1 Faculty of Chinese Medicine and State Key Laboratory of Quality Research in Chinese Medicine, Macau University of Science and Technology, Taipa, Macao PR China; 2 Zhuhai Hospital of Integrated Traditional Chinese and Western Medicine, Zhuhai, Guangdong Province 519000 PR China; 3 Joint Laboratory for Translational Cancer Research of Chinese Medicine of the Ministry of Education of the People’s Republic of China, Guangzhou University of Chinese Medicine, Guangzhou, 510006 PR China

**Keywords:** Acute toxicity, Improved up-and-down procedure, Median lethal dose, Modified Karber method, Nicotine, Sinomenine hydrochloride, Berberine hydrochloride

## Abstract

**Background:**

Up-and-down procedure (UDP) was recommended to replace traditional acute toxicity methods. However, it was limited due to the long experimental period (20–42 days). To improve UDP, an improved UDP method (iUDP) was developed by shortening observation time between sequence dosages. The aim of this study was to test the reliability of iUDP to provide a reliable method for the acute toxicity measurement of valuable or minor amount compounds.

**Methods:**

Oral median lethal dose (LD_50_) of nicotine, sinomenine hydrochloride and berberine hydrochloride were measured both by iUDP and modified Karber method (mKM).

**Results:**

LD_50_ of the three alkaloids measured by iUDP with 23 mice were 32.71 ± 7.46, 453.54 ± 104.59, 2954.93 ± 794.88 mg/kg, respectively. LD_50_ of the three alkaloids measured by mKM with 240 mice were 22.99 ± 3.01, 456.56 ± 53.38, 2825.53 ± 1212.92 mg/kg, respectively. The average time consumed by the two methods were 22 days and 14 days respectively. Total grams of the alkaloids used by the two methods were 0.0082 and 0.0673 (nicotine), 0.114 and 1.24 (sinomenine hydrochloride), 1.9 and 12.7 (berberine hydrochloride).

**Conclusion:**

iUDP could replace mKM to detect acute toxicity of substances with comparable and reliable result. And it is suitable for valuable or minor amount substances.

## Background

**Fig. 1:**
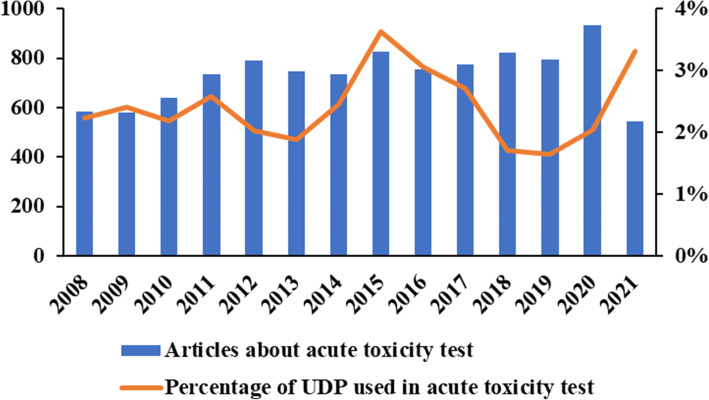
Percentage of UDP used in acute toxicity tests from January 2008 to August 2021

Median lethal dose (LD_50_) was first proposed by J. W. Trevan in 1976 [1]. It is used to study acute toxicity and classify toxic substance [2]. The 95% confidence interval (95% CI, μ ± σ) is used to describe LD_50_ mean [3, 4]. Traditional acute toxicity methods to detect LD_50_ and 95% CI include Bliss method [5, 6], modified Karber method (mKM) [7, 8], arithmetical method of Reed and Muench [9], and Miller and Tainter method [10]. For one substance, 50 ~ 80 mice would be administered to obtain LD_50_ in 14 days by mKM or other traditional methods (a 14-day observation would carry on survival animals) [11, 12]. In addition, the calculation of mKM is simple to obtain an accurately LD_50_ value and standard error. However, mKM violates animal rights and increase economic pressure [2, 13–15]. With 3Rs principles proposed (Reduction, Replacement, Refinement) [16, 17], up-and-down procedure (UDP) was advocated [14, 18]. In UDP, the dosage of (N + 1)^th^ would be determined by the poisoning symptoms of N^th^ animal after administration. Observed the N^th^ animal for 48 h, if it died, the dosage of (N + 1)^th^ would be reduced; Otherwise, dosage would be increased. It is particularly time-consuming to test acute toxicity of one compound by UDP using 4–15 animals (Different toxicity compounds show different death and survival reversals, which may take 20–42 days, Table 1). 10,259 journal articles about acute toxicity tests from January 2008 to August 2021 were analyzed by using SCI Finder. We found that UDP was employed in only 246 articles (Fig. 1). It is not ruled out that other alternatives are being used, but the low utilization rate of UDP is also noticeable. Low precision and long period are the two major factors that limit the popularity of UDP in acute toxicity studies [19–21]. Recently, several studies had gradually increased animal numbers to improve the usability of UDP [22–25]. In addition, Hiller, D.B. and Yu Y used UDP to detect drug intravenous toxicity. And they increased mice number at each dosage to improve precision of the results [26, 27]. Sarah C. Finch used UDP to test acute toxicity of tetrodotoxin and tetrodotoxin–saxitoxin mixtures under different routes (i.p. and p.o.) [28]. However, more animals mean more substances would be consumed which is not friendly to valuable or minor amount compounds. In this research, reducing observation time between sequence dosages rather than increasing animal number is applied to improve UDP. Nicotine, sinomenine hydrochloride and berberine hydrochloride, the three known toxic compounds are classic representatives of highly toxic, moderately toxic, and mildly toxic alkaloids. And they were poorly reported about oral acute toxicity of in mice [29, 30]. This study aimed to evaluate the feasibility and reliability of iUDP by comparing the LD_50_ of the three alkaloids tested both by iUDP and mKM.

**Table 1:** Comparison between UDP and traditional acute toxicity test methods

Method	Mice	Time (day)	Precision
UDP [31]	4 ~ 15	20 ~ 42	95% CI was wide, imprecise
Traditional acute toxicity methods			
Bliss method [5]	~ 80	14	95% CI was narrow, precise
mKM [32]	~ 80	14	95% CI was narrow, precise

## Materials and methods

### Experimental animals

A total of 263 ICR female mice (7 ~ 8-week-old, 26 ~ 30 g) were used. They were purchased from Beijing Vital River Laboratory Animal Technology Co., Ltd. The mice were housed in individually ventilated cages and had free access to food and water. A 12 h light/dark cycle was used in the room. The room temperature and humidity were 20 ~ 22 °C, 50 ~ 70%, respectively. Before the start of the study, the animal experiments were approved by the Division of Animal Control and Inspection, Department of Food and Animal Inspection and Control, Instituto para os Assuntos Cívicos e Municipais (IACM), Macao (AL020/DICV/SIS/2018).

In the experiment, each mouse was weighed and fasted 4 h with drink water freely before administration. For oral administration of nicotine and sinomenine hydrochloride, 0.2 ml was given for every 10 g of mice body weight. And 0.4 ml of berberine hydrochloride was given for every 10 g of mice body weight. After administration, the mice were fasted for 1 h with drink water freely. The survival or death of two consecutive animals is called reversal. For the main test, the testing stops when one of the following stopping criteria is occurred: (a) 3 subsequent animals survive at the highest dosage; (b) 5 reversals occur in any 6 subsequent animals administered; (c) at least 4 animals have followed the first reversal and the specified likelihood-ratios exceed the critical value.

When the experiment was stopped, all the survived mice were humanely killed and necropsied after a 14-day observation. Observed and recorded the pathological changes of organs.

### Materials

Nicotine (purity > 99%, CAS number: 54–11-5) and berberine hydrochloride (purity > 99%, CAS number: 2086-83-1) were obtained from Sigma Chemical Company (St. Louis, MO, USA). Sinomenine hydrochloride (purity > 99%, CAS number: 115–53-7) was kindly provided by Hunan Zhengqing Pharmaceutical Group Limited (Huaihua, Hunan Province, China).

### The acute toxicity assay of nicotine in mice by iUDP

According to previous literature results, nicotine was a highly toxic substance. Therefore, the estimated initial LD_50_ dosage was 20 mg/kg. Sigma was 0.2, slope was 5, and T was 1.6. Calculated the dosage by AOT425StatPgm. The sequential dosages were 2000, 1260, 800, 500, 320, 200, 126, 80, 50, 32, 20, 12.6, 8, 5, 3.2, 2 mg/kg. The first dosage of 12.6 mg/kg was given to the first mouse. Symptoms of poisoning were recorded within 24 h. If it was survived, 20 mg/kg was given as the second dosage. If it died, 8 mg/kg was chosen. Follow the experimental sequence until the standard stopping rules appeared.

### The acute toxicity assay of sinomenine hydrochloride in mice by iUDP

According to previous literature results, sinomenine hydrochloride was moderately toxic with a significant dosage-response relationship [30, 33]. Therefore, the estimated initial LD_50_ was 175 mg/kg. Sigma was 0.2, slope was 5, and T was 1.6. Calculated the dosage by AOT425StatPgm. The sequential dosages were 2000, 1100, 700, 440, 280, 175, 110, 70, 44, 28, 17.5, 11, 7, 4.4, 2.8, 1.75 mg/kg. The first dosage of 175 mg/kg was given to the first mouse. Symptoms of poisoning were recorded within 24 h. If it was survived, 280 mg/kg was given as the second dosage. If it died, 110 mg/kg was chosen. Follow the experimental sequence until the standard stopping rules appeared.

### The acute toxicity assay of berberine hydrochloride in mice by iUDP

According to previous literature results, berberine hydrochloride was a low or non-toxic compound. Therefore, the estimated initial LD_50_ dosage was 2500 mg/kg. Sigma was 0.5, slope was 2, and T was 3.16. Calculated the dosage by AOT425StatPgm. The sequential dosages were 5000, 2500, 790, 250, 79, 25, 7.9, 2.5, 0.79 mg/kg. The first dosage of 790 mg/kg was given to the first mouse. Symptoms of poisoning were recorded within 24 h. If it was survived, 2500 mg/kg was given as the second dosage. If it died, 250 mg/kg was chosen. Follow the experimental sequence until the standard stopping rules appeared.

### The acute toxicity assay of nicotine in mice by mKM

Twenty-four ICR female mice were randomly divided into 4 groups. The dosage ratio was 0.7, and oral dosage was 14, 20, 28.5, 40.8 mg/kg. The lowest dosage with 100% mortality (Dm = 40.8 mg/kg) and the highest dosage with 0% mortality (14 mg/kg) were obtained to provide references for subsequent experiments.

Fifty ICR female mice were randomly divided into 5 groups. The lowest and highest dosage were selected (16 mg/kg, 39.1 mg/kg, respectively). And 0.8 was chosen as the dosage ratio. After dosing, symptoms of poisoning, number of survival and dead mice were recorded. All mice were subjected to gross necropsy.

### The acute toxicity assay of sinomenine hydrochloride in mice by mKM

Twenty-four ICR female mice were randomly divided into 4 groups. The dosage ratio was 0.7, and oral dosage was 350, 500, 665, 715 mg/kg. Obtained the lowest dosage of 100% mortality (Dm = 665 mg/kg) and the highest dosage of 16% mortality (350 mg/kg). To obtain the highest dosage with 0% mortality (Dn), 300 mg/kg was added.

Fifty ICR female mice were randomly into 5 groups. The lowest and highest dosage were selected (300 mg/kg, 665 mg/kg, respectively). And 0.82 was chosen as the dosage ratio. After dosing, symptoms of poisoning, number of survival and dead mice were recorded. All mice were subjected to gross necropsy.

### The acute toxicity assay of berberine hydrochloride in mice by mKM

Twenty-four ICR female mice were randomly divided into 4 groups. The dosage ratio was 0.5, and oral dosage was 1000, 2000, 4000, 8000 mg/kg. The lowest dosage with 90% mortality (8000 mg/kg) and the highest dosage with 16.7% mortality (1000 mg/kg) were obtained. Then 11,428 (100% mortality) and 700 mg/kg (0% mortality) were carried out.

Fifty ICR female mice were randomly into 5 groups. The lowest and highest dosage were selected (703 mg/kg, 11,250 mg/kg, respectively). And 0.5 was chosen as the dosage ratio. After dosing, symptoms of poisoning, number of survival and dead mice were recorded. All mice were subjected to gross necropsy.

### Statistical analyses

In iUDP, the dosage and numbers of all survival and dead mice were recorded. The computational formula are as follows:

1 $$ {\mathrm{LD}}_{50}=\sum \left(\mathrm{Xi}\right)/\mathrm{N}+\left(\mathrm{A}+\mathrm{C}\right)\ast \mathrm{d}/\mathrm{N}, $$

2 $$ \mathrm{SE}=\mathrm{SD}\ast \surd \left(2/\mathrm{N}\right), $$

Wherein, Xi was the dosage level, N was the total number of animals, A and C values were obtained from Dixon’s tables [30], which were obtained from the number of O and X in N trials. And d was lgDn minus lgD(n + 1), SE was the standard error, SD was the standard deviation of all dosages in N trails.

In mKM, mortality rate of each group was calculated, and then values were substituted into formulas to obtain LD_50_ [34]. The computational formula are as follows:

3 $$ {\mathrm{LgLD}}_{50}=\mathrm{LgDmax}-\left(\mathrm{LgDN}-\mathrm{LgD}\left(\mathrm{N}+1\right)\right)\ \left(\sum \mathrm{p}-0.5\right), $$

4 $$ {\mathrm{SE}}_{50}=\mathrm{I}\ast \surd \left(\left(\sum \mathrm{p}-\sum \mathrm{p}\hat{\mkern6mu} 2\right)/\left(\mathrm{n}-1\right)\right), $$

5 $$ \mathrm{d}=\pm 4.5\ast {\mathrm{LD}}_{50}\ast {\mathrm{SE}}_{50}, $$

6 $$ \mathrm{CI}\ \mathrm{of}\ 95\%={\mathrm{LD}}_{50}\pm \mathrm{d}, $$

Wherein, m was LgLD_50_, D was the dosage of each group, Dmax was maximum dosage level, DN was the dosage of N group, D(N + 1) was the dosage of (N + 1) group, p was the mortality of each group of animals, and d was the standard error (σ), I was LgDN minus LgD (N + 1), and n was the number of animals in each group.

Data of organ indexes were plotted in GraphPad Prism (7.0) using One-way ANOVA and Dunnett’s multiple comparisons test. The data were presented in mean ± SD, *P < 0.05 vs Normal, **P < 0.01 vs Normal.

## Results

### The LD_50_ and toxicity of nicotine in mice detected by iUDP

The result was calculated as follows according to the results of Table 2 and formula (1), (2).

$$ {\mathrm{LD}}_{50}=228.6/7+\left(1.53+0.17\right)\ast 0.2/7=32.71, $$

$$ \mathrm{SE}=13.96\ast \surd 2/7=7.46, $$

**Table 2:** Lethality and signs of toxicity in mice administered with nicotine tested by iUDP

Seq.	Dosage (mg/kg)	Δm (g)	Short-term outcome	Symptoms	Pathology
1	12.6	1.1	O	Convulsive, weakness, recovered after 2 h	No visible alterations were found in organs and tissues
2	20	1.5	O	Violently convulsive, recovered after 2 h	Spleen was enlarged and congested
3	32	1.4	O	Violently convulsive, weakness, recovered after 6 h	Lung was enlarged and congested
4	50	0.9	X	Violently convulsive, dead after 5 min	Heart and lung were enlarged
5	32	1.1	O	Violently convulsive, weakness, recovered after 6 h	Heart and lung were markedly enlarged
6	50	1.7	X	Violently convulsive, dead after 10 min	Heart, liver and lung were enlarged
7	32	1.4	X	Violently convulsive, dead after 5 min	Heart, liver and lung were enlarged
Stop criteria met: 3 reversals in 5 tests					

*Note*: The sequence of outcomes: O for alive and X for dead

Therefore, the LD_50_ for nicotine was 32.71 mg/kg and the 95% CI was [25.25, 40.17].

Compared with normal mice, lung in mice administered with different dosage of nicotine were enlarged (Table 3). There was a good dosage-effect relationship of nicotine on lung injury in mice. As seen in Tables 3, 32 mg/kg of nicotine increased lung weight in mice (*P* = 0.007). And 50 mg/kg of nicotine significantly increased heart and lung weight in mice (*P* = 0.009, *P* = 0.010).

**Table 3:** Effect of nicotine on organ indexes in ICR mice by iUDP

Dosage (mg/kg)	Heart (%)	Liver (%)	Spleen (%)	Lung (%)	Kidney (%)
0	0.466 ± 0.002	4.800 ± 0.373	0.387 ± 0.079	0.588 ± 0.057	1.282 ± 0.140
12.6	0.491	4.665	0.370	0.609	1.248
20	0.485	4.250	0.381	0.643	1.185
32	0.474 ± 0.018	4.548 ± 0.505	0.366 ± 0.084	0.653 ± 0.056**	1.170 ± 0.058
50	0.581 ± 0.051**	5.123 ± 0.155	0.385 ± 0.063	0.702 ± 0.015**	1.107 ± 0.007

*Note*: *P < 0.05 vs Normal, **P < 0.01 vs Normal

### The LD_50_ and toxicity of sinomenine hydrochloride in mice detected by iUDP

The result was calculated as follows according to the results of Table 4 and formula (1), (2).

$$ {\mathrm{LD}}_{50}=3175/7+\left(1.53+0.16\right)\ast 0.2/7=453.54, $$

$$ \mathrm{SE}=195.67\ast \surd 2/7=104.59, $$

**Table 4:** Lethality and signs of toxicity of mice administered with sinomenine hydrochloride by iUDP

Seq.	Dosage (mg/kg)	Δm (g)	Short-term outcome	Symptoms	Pathology
1	175	1.1	O	Mild, shortness of breath, frightened, recovered after 2 h	No visible alterations were found in organs
2	280	1.4	O	Shortness of breath, frightened, recovered after 5 h	No visible alterations were found in organs
3	440	1.8	O	Tremor, breathlessness, and recovered after 2 h	Liver was enlarged
4	700	1.3	X	Severe tremor, weakness, dead after 30 min	Liver was enlarged
5	440	1.5	O	Mild tremor, weakness, and recovered after 2 h	Liver and kidney were enlarged
6	700	0.9	X	Severe tremor, weakness, dead after 1 h	Liver was enlarged
7	440	0.9	X	Breathlessness, tremor, and dead after 4 h	Liver and kidney were enlarged
Stop criteria met: 5 reversals in 6 tests					

*Note*: The sequence of outcomes: O for alive and X for dead

Therefore, the LD_50_ of sinomenine hydrochloride was 453.54 mg/kg and the 95% CI was [349.0, 558.2].

Compared with normal mice, sinomenine hydrochloride has no effect on the organ indexes (Table 5). No visible alterations were found in organs and tissues in mice administered with low dosage of sinomenine hydrochloride. 700 mg/kg of sinomenine hydrochloride significantly increased heart, spleen and kidney weight in mice by comparison with normal mice (*P* = 0.010, *P* = 0.001, *P* = 0.007).

**Table 5:** Effect of sinomenine hydrochloride on organ indexes in ICR mice by iUDP

Dosage (mg/kg)	Heart (%)	Liver (%)	Spleen (%)	Lung (%)	Kidney (%)
0	0.466 ± 0.002	4.800 ± 0.373	0.387 ± 0.079	0.588 ± 0.057	1.282 ± 0.140
175	0.550	4.660	0.312	0.623	1.120
280	0.450	4.258	0.467	0.578	1.295
440	0.403 ± 0.012	4.382 ± 0.442	0.345 ± 0.082	0.519 ± 0.110	1.110 ± 0.035*
700	0.315 ± 0.065**	4.452 ± 0.486	0.293 ± 0.033**	0.566 ± 0.065	1.005 ± 0.085**

*Note*: *P < 0.05 vs Normal, **P < 0.01 vs Normal

### The LD_50_ and toxicity of berberine hydrochloride in mice detected by iUDP

The result was calculated as follows according to the results of Table 6 and formula (1), (2).

$$ {\mathrm{LD}}_{50}=26580/9+\left(1.53+0.16\right)\ast 0.2/9=2954.93, $$

$$ \mathrm{SE}=1686.29\ast \surd \left(2/9\right)=794.88, $$

**Table 6:** Lethality and signs of toxicity of mice administrated with berberine hydrochloride by iUDP

Seq.	Dosage (mg/kg)	Δm (g)	Short-term outcome	Symptoms	Pathology
1	790	1.1	O	Reduced activity, recovered after 2 h	No visible alterations were found in organs and tissues
2	2500	1.5	O	Reduced activity, recovered after 4.5 h	No visible alterations were found in organs and tissues
3	5000	1.4	X	Reduced activity, weakness, dead after 10 h	Liver was congested
4	2500	0.9	O	Reduced activity, recovered after 4.5 h	No visible alterations were found in organs and tissues
5	5000	1.1	X	Reduced activity, weakness, dead after 8 h	Liver was congested
6	2500	1.7	X	Reduced activity, dead after 16 h	No visible alterations were found in organs and tissues
7	790	1.4	O	Reduced activity, recovered after 1 h	No visible alterations were found in organs and tissues
8	2500	1.1	O	Reduced activity, recovered after 4 h	No visible alterations were found in organs and tissues
9	5000	1.0	X	Reduced activity, weakness, and dead after 18 h	Liver was congested
Stop criteria met: 3 reversals in 5 tests					

*Note*: The sequence of outcomes: O for alive and X for dead

Therefore, the LD_50_ of berberine hydrochloride was 2954.93 mg/kg and the 95% CI was [2160.05, 3749.81].

Compared with normal mice, 5000 mg/kg of berberine hydrochloride increased spleen weight in mice (*P* = 0.049, Table 7). No visible alterations were found in organs and tissues in mice administered with berberine hydrochloride.

**Table 7:** Effect of berberine hydrochloride on organ indexes in ICR mice by iUDP

Dosage (mg/kg)	Heart (%)	Liver (%)	Spleen (%)	Lung (%)	Kidney (%)
0	0.466 ± 0.002	4.800 ± 0.373	0.387 ± 0.079	0.588 ± 0.057	1.282 ± 0.140
790	0.472 ± 0.028	4.602 ± 0.295	0.363 ± 0.063	0.580 ± 0.097	1.100 ± 0.100
2500	0.449 ± 0.045	4.472 ± 0.207	0.427 ± 0.096	0.627 ± 0.108	1.280 ± 0.073
5000	0.465 ± 0.039	4. 503 ± 0.200	0.426 ± 0.041*	0.598 ± 0.049	1.129 ± 0.068

*Note*: *P < 0.05 vs Normal, **P < 0.01 vs Normal

### The LD_50_ and toxicity of nicotine in mice detected by mKM

The result was calculated as follows according to Table 8 and formula (3, 4, 5, 6).

$$ {\mathrm{LgLD}}_{50}=\lg 39.1-\left(\lg 20-\lg 16\right)\ast \left[2.9-0.5\right]=1.3616, $$

$$ {\mathrm{LD}}_{50}=22.99, $$

$$ {\mathrm{SE}}_{50}=0.096\ast \surd \left(\left(2.9-2.07\right)/\left(10-1\right)\right)=0.02915, $$

$$ \mathrm{SE}=\pm 4.5\ast 22.99\ast 0.02915=3.02, $$

**Table 8:** Lethality and signs of toxicity of mice administrated with nicotine by mKM

Group	n	Dosage (mg/kg)	Morality (p)	p2	Pathology
1	10	16	0.2	0.04	No visible alterations were found in other organs and tissues.
2	10	20	0.3	0.09	Liver was enlarged and congested
3	10	25	0.7	0.49	Liver was enlarged and congested
4	10	31.25	0.8	0.64	Liver and kidney were enlarged and congested
5	10	39.1	0.9	0.81	Liver and kidney were significantly enlarged and congested

*Note*: The sequence of outcomes: O for alive and X for dead

Therefore, the LD_50_ of nicotine was 22.99 mg/kg and the 95% CI was [19.97, 26.01].

Compared with normal mice, 25 and 31.25 mg/kg of nicotine increased lung weight in mice (*P* = 0.024, *P* = 0.009, respectively). 39.10 mg/kg of nicotine significantly increased lung weight in mice (*P* = 0.005, Table 9).

**Table 9:** Effect of different doses of nicotine on organ indexes in ICR mice by mKM

Dosage (mg/kg)	Heart (%)	Liver (%)	Spleen (%)	Lung (%)	Kidney (%)
0	0.466 ± 0.002	4.800 ± 0.373	0.387 ± 0.079	0.588 ± 0.057	1.282 ± 0.140
16	0.467 ± 0.023	4.667 ± 0.317	0.412 ± 0.066	0.603 ± 0.046	1.177 ± 0.075
20	0.482 ± 0.061	4.772 ± 0.476	0.468 ± 0.068	0.603 ± 0.081	1.220 ± 0.064
25	0.431 ± 0.002	4.825 ± 0.034	0.578 ± 0.154	0.665 ± 0.038*	1.211 ± 0.021
31.25	0.437 ± 0.009	4.272 ± 0.363	0.423 ± 0.022	0.692 ± 0.058**	1.187 ± 0.052
39.10	0.490 ± 0.041	4.891 ± 0.105	0.391 ± 0.055	0.700 ± 0.020**	1.137 ± 0.09

*Note*: *P < 0.05 vs Normal, **P < 0.01 vs Normal

### The LD_50_ and toxicity of sinomenine hydrochloride in mice detected by mKM

The result was calculated as follows according to Table 10 and formula (3, 4, 5, 6).

$$ {\mathrm{LgLD}}_{50}=\lg\ 663-\left(\lg 300-\lg 365\right)\ast \left[2.3-0.5\right]=2.66, $$

$$ {\mathrm{LD}}_{50}=456.56, $$

$$ {\mathrm{SE}}_{50}=0.09\ast \surd \left(\left(2.3-1.55\right)/\left(10-1\right)\right)=0.02598, $$

$$ \mathrm{SE}=\pm 4.5\ast 456.56\ast 0.02598=53.38, $$

**Table 10:** Lethality and signs of toxicity of mice administrated with sinomenine hydrochloride by mKM

Group	n	Dosage (mg/kg)	Morality (p)	p2	Pathology
1	10	300	0	0	No visible alterations were found in organs and tissues
2	10	365	0.3	0.09	Liver was enlarged and congested
3	10	446	0.4	0.16	Liver was enlarged and congested
4	10	544	0.7	0.49	Liver and kidney were enlarged and congested
5	10	663	0.9	0.81	Liver and kidney were significantly enlarged and congested

Therefore, the LD_50_ of sinomenine hydrochloride was 456.56 mg/kg and he 95% CI was [403.18, 509.94].

Compared with normal mice, the heart and kidney in mice administered by 665 mg/kg of sinomenine hydrochloride were enlarged (*P* = 0.035, *P* = 0.003, respectively, Table 11).

**Table 11:** Effect of different doses of sinomenine hydrochloride on organ indexes in ICR mice by mKM

Dosage (mg/kg)	Heart (%)	Liver (%)	Spleen (%)	Lung (%)	Kidney (%)
0	0.466 ± 0.002	4.800 ± 0.373	0.387 ± 0.079	0.588 ± 0.057	1.282 ± 0.140
300	0.494 ± 0.091	4.948 ± 0.500	0.404 ± 0.085	0.571 ± 0.109	1.217 ± 0.184
365	0.454 ± 0.036	4.925 ± 0.298	0.393 ± 0.063	0.586 ± 0.092	1.101 ± 0.104
446	0.403 ± 0.012	4.382 ± 0.442	0.335 ± 0.082	0.519 ± 0.110	1.210 ± 0.035
544	0.421 ± 0.037	3.931 ± 0.240	0.327 ± 0.078	0.543 ± 0.022	1.109 ± 0.110*
663	0.345 ± 0.035**	4.327 ± 0.248	0.305 ± 0.021	0.554 ± 0.054	0.973 ± 0.063**

*Note*: *P < 0.05 vs Normal, **P < 0.01 vs Normal

### The LD_50_ and toxicity of berberine hydrochloride in mice detected by mKM

The result was calculated as follows according to Table 12 and formula (3, 4, 5, 6).

$$ {\mathrm{LgLD}}_{50}=\lg\ 11250-\left(\lg 1406-\lg 703\right)\ast \left[2.5-0.5\right]=3.4511, $$

$$ {\mathrm{LD}}_{50}=2825.53, $$

$$ {\mathrm{SE}}_{50}=0.3\ast \surd \left(\left(2.5-1.59\right)/\left(10-1\right)\right)=0.09539, $$

$$ \mathrm{SE}=\pm 4.5\ast 2825.53\ast 0.09539=1212.92, $$

**Table 12:** Lethality and signs of toxicity of mice administered with berberine hydrochloride by mKM

Group	n	Dosage (mg/kg)	Morality (p)	p2	Pathology
1	10	703	0.2	0.04	No visible alterations were found in other organs and tissues
2	10	1406	0.3	0.09	No visible alterations were found in other organs and tissues
3	10	2812	0.4	0.16	No visible alterations were found in other organs and tissues
4	10	5628	0.7	0.49	Lung were enlarged
5	10	11,250	0.9	0.81	Liver and lung were enlarged, and spleen was reduced

Therefore, the LD_50_ of berberine hydrochloride was 2825.53 mg/kg and the 95% CI was [1612.60, 4038.45].

Compared with normal mice, the liver, spleen and lung in mice administered by 11,250 mg/kg of berberine hydrochloride were enlarged (*P* = 0.002, *P* = 0.009, *P* = 0.01, respectively Table 13).

**Table 13:** Effect of berberine hydrochloride on organ indexes in ICR mice by mKM

Dosage (mg/kg)	Heart (%)	Liver (%)	Spleen (%)	Lung (%)	Kidney (%)
0	0.466 ± 0.002	4.800 ± 0.373	0.387 ± 0.079	0.588 ± 0.057	1.282 ± 0.140
703	0.463 ± 0.018	5.010 ± 0.558	0.406 ± 0.092	0.553 ± 0.069	1.227 ± 0.203
1406	0.429 ± 0.028	4.740 ± 0.295	0.422 ± 0.063	0.645 ± 0.097	1.162 ± 0.100
2812	0.454 ± 0.017	4.453 ± 0.242	0.398 ± 0.075	0.667 ± 0.031	1.198 ± 0.131
5628	0.473 ± 0.046	4.575 ± 0.173	0.394 ± 0.042	0.625 ± 0.024	1.320 ± 0.073
11,250	0.442 ± 0.053	5.877 ± 0.309**	0.288 ± 0.065**	0.697 ± 0.090**	1.249 ± 0.110

*Note*: *P < 0.05 vs Normal, **P < 0.01 vs Normal

## Discussion

We have improved UDP for acute toxicity testing of substances. The improved UDP (iUDP) has several advantages. It shortens the experiment period to improve the usability of UDP. Besides, iUDP is very friendly to valuable or minor amount substances. Different kinds of new natural products or monomers from Traditional Chinese Medicine or herbal medicine, often with low yield or high cost. To confirm the safety of such compounds, iUDP is a viable option. However, what cannot be ignored is that oral LD_50_ is affected by many factors such as gender, age and fasting time, etc. [2]. Gender differences plays an important role in dose-effect response [35, 36]. Females are more sensitive to compound than males [37]. It is recommended to use females for general acute toxicity studies [33]. Age, which is often poorly reported, affects the physiological state and sensitivity to substance [38]. Four to eight weeks mice (18 ~ 30 g) are often used in toxicity tests [39–42]. It is indicated that ICR, KM, and BALB/c mice (26 ~ 30 g) under the state of 8 ~ 10 weeks are equivalent to the human adulthood [43]. To increase scientific validity and reduce experimental variability, the adult rodent animals are used in acute toxicity experiments [44]. In addition, the fasting status is often overlooked. It was reported that overnight-fasting affected the level of hormone and sensitivity of animals to drugs [45]. In this study, a 4 h-fasting is recommended for mice.

According to toxicity categories in Classification Criteria for Acute Toxicity (Table 14) [46] and LD_50_ results (Table 15), nicotine, sinomenine hydrochloride and berberine hydrochloride were divided into Category II (Toxicity), IV (Mildly toxicity) and V (Low toxicity). Consequently, we believe that compounds with the same or similar toxicity as these three alkaloids can be tested by iUDP. However, iUDP is not suitable for acute toxicity test of completely non-toxic compound, which is also the defect caused by shortening the observation interval time to 24 h. In the experiment, surviving mice returned to normal after 2 ~ 18 h administration (Tables 2, 4, 6). Nicotine and sinomenine hydrochloride have a fast-poisoning reaction which was relieve within 4–6 h. But unknown chemicals may take a longer time to show its toxic reaction which is the same as berberine hydrochloride (dead after administration of 8-18 h). To improve the repeatability of iUDP, the state of each animal should be as consistent as possible to reduce individual differences of animals [2, 47, 48]. It is best to fix the fasting start time and end time for each mouse. In this article, the mice were fasted daily from 9:00 am to 13:00 pm and the weight loss of each mouse was between 0.9 to 2.0 g.

**Table 14:** Classification Criteria for Acute Toxicity [46]

Exposure route	Category I Highest toxicity	Category II Toxicity	Category III Moderately toxicity	Category IV Mildly toxicity	Category V Low acute toxicity
Oral (mg/kg)	<5	5 ~ 50	51 ~ 300	301 ~ 2000	2001 ~ 5000

**Table 15:** Comparison of acute toxicity results between iUDP and mKM in three alkaloids

Method	Compound	Category	Animals	Compound (g)	Expense (MOP)	Duration (Day)
iUDP	Nicotine	II	7	0.0082	1330	21
	Sinomenine hydrochloride	IV	7	0.114	1330	21
	Berberine hydrochloride	V	9	1.9	1900	24
mKM	Nicotine	II	74	0.0673	14,060	14
	Sinomenine hydrochloride	IV	80	1.24	15,200	14
	Berberine hydrochloride	V	86	12.7	16,340	14

In addition, the reliability and accuracy of iUDP could be improved by choosing appropriate initial dosage and slope. Initial dosage should be valued from all known toxicity information [49]. Slope of dosage response curve is a key regulator for sequential dosage. A larger slope would bring a good 95%CI, which may lead to increase animal. A smaller slope would reduce the accuracy of 95%CI. Once the slope setting is not suitable, the entire experiment faced the risk of failure.

## Conclusion

In light of experimental results, it may be concluded that iUDP is reliable to detect acute toxicity of unknown substances. Compared with traditional acute toxicity method, iUDP is more animal-friendly and economy and therefore suitable for valuable or minor amount substances.

## Data Availability

All data generated or analyzed during this study are included in this published article.

## Abbreviations

95% CI: 95% confidence interval
iUDP: Improved up-and-down procedure
LD_50_: Median lethal dose
mKM: Modified Karber method
